# Xinbao Pill ameliorates heart failure via regulating the SGLT1/AMPK/PPARα axis to improve myocardial fatty acid energy metabolism

**DOI:** 10.1186/s13020-024-00959-1

**Published:** 2024-06-11

**Authors:** Linjie Pan, Zhanchi Xu, Min Wen, Minghui Li, Dongxin Lyu, Haiming Xiao, Zhuoming Li, Junhui Xiao, Yuanyuan Cheng, Heqing Huang

**Affiliations:** 1 School of Pharmaceutical Sciences, Sun Yat-sen University, Guangzhou, 510006 China; 2 Guangzhou Hospital of Integrated Traditional and Western Medicine, 87 Yingbin Road, Guangzhou, 510801 China; 3 School of Pharmaceutical Sciences, Guangzhou University of Chinese Medicine, Guangzhou, 510006 China

**Keywords:** Xinbao Pill, Heart failure, Myocardial energy metabolism, SGLT1, AMPK/PPARα axis

## Abstract

**Background:**

Heart failure (HF) is characterized by a disorder of cardiomyocyte energy metabolism. Xinbao Pill (XBW), a traditional Chinese medicine formulation integrating “Liushen Pill” and “Shenfu Decoction,” has been approved by China Food and Drug Administration for the treatment of HF for many years. The present study reveals a novel mechanism of XBW in HF through modulation of cardiac energy metabolism.

**Methods:**

In vivo, XBW (60, 90, 120 mg/kg/d) and fenofibrate (100 mg/kg/d) were treated for six weeks in *Sprague–Dawley* rats that were stimulated by isoproterenol to induce HF. Cardiac function parameters were measured by echocardiography, and cardiac pathological changes were assessed using H&E, Masson, and WGA staining. In vitro, primary cultured neonatal rat cardiomyocytes (NRCMs) were induced by isoproterenol to investigate the effects of XBW on myocardial cell damage, mitochondrial function and fatty acid energy metabolism. The involvement of the SGLT1/AMPK/PPARα signalling axis was investigated.

**Results:**

In both in vitro and in vivo models of ISO-induced HF, XBW significantly ameliorated cardiac hypertrophy cardiac fibrosis, and improved cardiac function. Significantly, XBW improved cardiac fatty acid metabolism and mitigated mitochondrial damage. Mechanistically, XBW effectively suppressed the expression of SGLT1 protein while upregulating the phosphorylation level of AMPK, ultimately facilitating the nuclear translocation of PPARα and enhancing its transcriptional activity. Knockdown of SGLT1 further enhanced cardiac energy metabolism by XBW, while overexpression of SGLT1 reversed the cardio-protective effect of XBW, highlighting that SGLT1 is probably a critical target of XBW in the regulation of cardiac fatty acid metabolism.

**Conclusions:**

XBW improves cardiac fatty acid energy metabolism to alleviate HF via SGLT1/AMPK/PPARα signalling axis.

**Supplementary Information:**

The online version contains supplementary material available at 10.1186/s13020-024-00959-1.

## Introduction

Heart failure (HF) frequently manifests in the advanced stages of various cardiovascular diseases and is characterized by ventricular hypertrophy, myocardial fibrosis, and disturbances in myocardial energy metabolism [1]. Recently, the prevalence of HF has increased due to the ageing of the population [2]. Disruption in myocardial energy metabolism is the underlying cause of diminished myocardial energy supply, thereby expediting disease progression [3]. In a healthy heart, fatty acid (FA) metabolism plays a dominant role in providing energy, with its substantial production of ATP making the heart an essential hub for energy generation within the body [4]. However, in the failing heart, there is an energy shift metabolic preference characterized by reduced fatty acid oxidation (FAO) and increased reliance on glucose [5]. While this change may decrease oxygen consumption, glycolysis produces far less ATP than fatty acid oxidation, which fails to meet the energetic demands of the heart. Therefore, increasing the overall energy level of the heart is a key strategy for the treatment of HF.

Currently, novel strategies have emerged in managing HF that focus on increasing energy supply and counteracting metabolic remodelling [5, 6]. Numerous studies have proven that activation of the AMPK/PPARα signalling pathway prevents the development of HF by increasing cardiac fatty acid metabolism [7]. AMPK acts as a “switch” of cellular energy metabolism, capable of self-activation in response to changes in the intracellular AMP/ATP ratio [8]. As a result, AMPK is of great importance in maintaining energy balance, mitochondrial function, and autoimmunity [9]. The activation of phosphorylated AMPK enhances the transcriptional activity of PPARα [10, 11]. As a nuclear receptor, PPARα plays a pivotal role in diverse biological processes and influences myocardial energy metabolism by modulating genes involved in FAO [12, 13].

Traditional Chinese Medicine (TCM) has demonstrated efficacy in the clinical management of HF, exhibiting the characteristics of being multi-component and multi-targeted [14, 15]. Xinbao Pill (XBW) is a TCM compound medication that exhibits significant clinical efficacy in treating HF, angina, and arrhythmia [16]. According to the theory of TCM, it possesses the functions of “warming and tonifying the heart and kidneys,” “benefiting qi and reinforcing yang,” and “promoting blood circulation and regulating collaterals.” Therefore, it has been approved by the China Food and Drug Administration as a TCM remedy for treating clinical cardiovascular diseases, with the production approval number Z44021843. The formula contains nine ingredients: *Datura metel* L. (common name: Yangjinhua in China, YJH), *Panax ginseng* C.A.Mey. (Renshen, RS), *Panax notoginseng* (Burk.) F.H.Chen (Sanqi, SQ), *Cinnamomum cassia* Presl (Rougui, RG), *Aconitum carmichaelii* Debx (Fuzi, FZ), *Cervi Cornu Pantotrichum* (Lurong, LR), *Bufonis Venenum* (Chansu, CS), *Borneolum syntheticum* (Bingpian, BP) and *Moschus moschiferus* (Shexiang, SX) (Table 1; the names of the ingredients were obtained from https://herb.ac.cn/ and https://www.worldfloraonline.org/) [17]. Pharmacological studies showed that XBW reduces cardiac hypertrophy and improves cardiac function by inhibiting phosphorylation of PI3K/Akt signalling as well as GSK3β [18]. Moreover, XBW has been discovered to possess a curative impact on myocardial ischemia–reperfusion injury and ischemic cardiac failure [19].

**Table 1:** The composition of XBW

No	Chinese name	Latin name	Part used or Source
1	Yangjinhua	*Datura metel* L	Flower
2	Renshen	*Panax ginseng* C.A.Mey	Root and rhizome
3	Sanqi	*Panax notoginseng* (Burk.) F.H.Chen	Root and rhizome
4	Rougui	*Cinnamomum cassia* Presl	Bark
5	Fuzi	*Aconitum carmichaelii* Debx	Lateral root
6	Lurong	*Cervi Cornu Pantotrichum*	Non-ossifying young horn of *Cervus nippon* Temminck
7	Chansu	*Bufo bufo gargarizans*	Dried secretion from skin glands of *Bufo bufo gargarizans* Cantor
8	Bingpian	*Borneolum*	Resin
9	Shexiang	*Moschus moschiferus*	Dried secretions from the glands of the male *Moschus moschiferus* L

In this study, we identified that the cardiac-protective role of XBW depends on regulating myocardial energy metabolism. XBW could promote the activation of the AMPK/PPARα axis by downregulating SGLT1 expression, restoring cardiac FA energy metabolism, and effectively ameliorating HF.

## Materials and methods

### Materials and reagents

XBW was provided by Guangdong Xinbao Pharmaceutical Technology Co., LTD. (Guangzhou, China). The composition profile, chemical composition identification, and quality control of XBW were previously reported in our previous study [19]. The β-MHC antibody (M8421) and Isoprenaline hydrochloride (I5627, for in vivo experiments) were acquired from Sigma-Aldrich Corporation (St. Louis, MO, USA). Isoprenaline hydrochloride (HY-B0468, for in vitro experiments) was procured from MedChemExpress (New Jersey, USA). Antibodies against AMPK (AF6423), p-AMPK (AF3423), SGLT1 (DF7202), PPARα (AF5301), ANP (6497), CD36 (DF13262) were purchased from Affinity Biosciences (Changzhou, China). Antibodies against α-Tubulin (11,224-1-AP), FN (15,613-1-AP), α-SMA (14,395-1-AP), ACADM (55,210-1-AP), CPT1-1B (22,170-1-AP), NRF1 (12,482-1-AP), PGC1α (66,369-1-IG) were purchased from Proteintech Group (Wuhan, China).

### Cell culture and drug administration

The extraction and culture methods of NRCMs were conducted following established protocols [20]. All actions were carried out following the Animal Welfare Law of China and were authorized by the Ethics Committee of Sun Yat-sen University. The hearts of neonatal rats (1–3 days old) were aseptically collected using ophthalmic forceps, dissected into small fragments, and enzymatically digested with PBS containing 0.8% trypsin. Following multiple rounds of centrifugation, the supernatant was carefully removed, while the cardiomyocytes were selectively retained through differential centrifugation. After a 24 h incubation period, the culture medium was refreshed with fresh medium supplemented with 20% FBS, and the cells were subsequently placed in a CO_2_ incubator set at 37 °C for further cultivation.

### Animal experiments

The Ethics Committee of Guangzhou University of Chinese Medicine approved the animal study’s experimental procedures and protocols. The male *Sprague–Dawley* rats, aged 6–8 weeks and in good health, were procured from the Laboratory Animal Center of Sun Yat-sen University. The average body weight was approximately 230 g, with a fluctuation range of ± 10 g. After seven days of adaptive feeding, the rats were randomly divided into two groups: the Normal group consisted of 7 SD rats selected at random, while the remaining 56 rats received subcutaneous injections of ISO (5 mg/kg/day) for seven consecutive days to establish the HF model. During this period, the rats in the Normal group also received an equal volume of saline administered similarly. After three weeks of regular feeding, echocardiography was used to verify the expected effect of HF modelling. HF in rats was considered successfully induced when the left ventricular ejection fraction (LVEF) value was below 65%. Subsequently, 35 rats were randomly selected from the successfully established HF model rats and then divided into five experimental groups: the HF group, XBW low dose (XBW-L) group (60 mg/kg/day), XBW medium dose (XBW-M) group (90 mg/kg/day), XBW high dose (XBW-H) group (120 mg/kg/day), and the positive drug fenofibrate (Feno) group (100 mg/kg/day). The treatment groups were administered XBW and Feno, respectively, while the normal group and HF group received an equal volume of 0.5% CMC-Na solution as a negative control. The treatments were orally administered via gavage for six consecutive weeks.

### Echocardiographic measurements

After the administration, isoflurane was administered to induce anaesthesia in rats. Afterwards, a detection probe was placed on the left side of the ribs within the chest to evaluate the heart’s performance. Echocardiography was performed using the Vevo2100 high-resolution small animal ultrasound imaging system by capturing a short-axis section of the left ventricle.

### Analysis of serum biomarkers

CK-MB assay kit (H197-1-1), LDH assay kit (A020-2-2), and AST assay kit(C010-2-1) were purchased from Nanjing Jiancheng Bioengineering Institute (Nanjing, China). BNP Elisa Kit (CSB-E07972R) and cTn-I Elisa kit (CSB-E08594r) were obtained from Cusabio Corporation (Wuhan, China). All biochemical parameters were measured using the respective kits following the related instructions.

### Pathological sections and immunohistochemistry

Sections of paraffin-embedded hearts, measuring four μm, were prepared for staining with HE, Masson, and WGA. The myocardial expressions of ANP, CPT1B, SGLT1, and PPARα in heart tissue were detected by immunohistochemistry. EVOS FL Auto captured all the images.

### MTT assay

The NRCMs were placed in 96-well dishes and cultured with XBW at various concentrations, ranging from 2 to 128 μg/mL. The wells were incubated at 37 °C for an additional four hours following 24 h, during which a solution of MTT (5 mg/mL) was introduced. Then, the wells were treated with DMSO (200 μL). BCA levels were measured using a microplate reader set to a wavelength of 490 nm after gentle shaking induced colour change.

### Cell surface area assay

After XBW treatment, NRCMs were fixed with 4% paraformaldehyde (PA) and lysed with 0.3% Triton X-100 at room temperature. Subsequently, cells were thoroughly rinsed with PBS and stained using Rhodamine-phalloidin in combination with DAPI staining solution. Finally, images were promptly acquired utilizing the EVOS cell imaging system.

### Western blot (WB) assay

After the experiments, the cells or myocardial tissue were lysed by utilizing 1 × RIPA buffer that included protease inhibitors and phosphatase inhibitors. After determining the protein concentration, the samples were electrophoresed on a gel made of 10% SDS–polyacrylamide. The proteins were subsequently transferred onto PVDF membranes, which were then sealed with 5% skim milk for 1 to 2 h, followed by a washing step using TBST. Specific primary antibodies (ANP, β-MHC, FN, α-SMA, SGLT1, AMPK, PAMPK, PPARα, ACADM, CD36, CPT-1B, PGC1α, NRF1, α-Tubulin) were incubated overnight. Following incubation, the membranes were subjected to a secondary antibody labelled with HRP at ambient temperature for 1 h. Following rinsing with TBST, the images were automatically captured using an ECL chemiluminescence imaging system and ECL chemiluminescence solution.

### Network pharmacological assay

The drugs’ potential targets were sourced from Herb, ETCM (http://www.tcmip.cn/ETCM/), and TCMSP (https://tcmsp-e.com/). The disease targets of HF were acquired from the Genecards (https://www.genecards.org/ds/) [21] and DisGeNET databases (https://www.disgenet.org/) [22]. The drug-disease cross-target set was visualized using a Venn diagram. The PPI network of key targets was established and analyzed using the String11.0 (https://string-db.org/) database and Cytoscape 3.8.2 software (San Diego, California, USA). Finally, Gene Ontology (GO) and Kyoto Encyclopedia of Genes and Genomes (KEGG) enrichment analyses were performed on the potential targets to predict the underlying biological processes and signalling pathways involved in XBW’s treatment of HF.

### Immunofluorescence (IF) assay

The cells were seeded in Confocal glass bottom dishes. After administration, the cells were rinsed with PBS and preserved in their original morphology using a 4% PA, while the cell membrane was disrupted by treatment with 0.3% Triton X-100. Subsequently, the cells were blocked with goat serum, followed by incubation with primary antibody in goat serum and staining with specific secondary antibody along with DAPI staining solution. Finally, the stained cells were promptly observed under a Zessie LSM 510 laser confocal microscope for image acquisition.

### Dual luciferase assay

After adhering to the plates, the NRCMs cells were seeded in 96-well opaque plates and transfected with 0.2 μg of HA-PPARα, pGMPPAR-Lu, and pRL-TK. After 24 h, the cells underwent fluid exchange followed by treatment with ISO to induce NRCM hypertrophy and XBW drug for 24 h. Luciferase activity was measured to obtain gene activation results using the Dual-Luciferase Reporter Assay System kit.

### XF cell mito stress test

The XF Cell Mito Stress Test is an effective approach to quantifying intracellular energy levels by assessing the oxygen consumption rate (OCR) in viable cells using the Seahorse XF Energy Metabolism Detection System to evaluate mitochondrial function. The experimental steps are as follows: NRCMs were seeded into Seahorse XF 96-cell plates and then subjected to specific experimental treatments. Simultaneously, the Seahorse probe plate was hydrated at 37 °C in a non-CO_2_ incubator. On the day of the experiment, the detection solution and drug solution were prepared according to the kit instructions, and the cell DMEM culture medium was replaced with the detection solution. The experiment was performed using the Seahorse XF instrument according to established protocols.

### Mitochondrial staining assay

The cells were seeded onto Confocal glass bottom dishes. Following the administration of the drug, the medium was substituted with a DMEM medium that included a JC-1 working solution. This was followed by a 20 min incubation at 37 ℃ without light. Following incubation, the supernatant was washed with JC-1 staining buffer, and fresh medium was added for observation using a laser confocal microscope and image collection. After administering the drug, cells were washed with PBS and then incubated in the dark for 20 min with a Mito-Tracker working solution in serum-free DMEM medium for Mito-Tracker staining. Subsequently, fresh serum-free DMEM medium was added at room temperature before observation under a laser confocal microscope and image collection.

### Plasmid transfection

The SGLT1 plasmid was synthesized by YouBio (Changsha, China). Once the NRCMs cells reached optimal growth, 5 μg of the plasmid was transfected into NRCMs using MIKX transfection efficiency adjuvant. After 24 h, the fresh medium was replaced, and drug administration commenced.

### Small interfering RNA and transient transfection

The SGLT1 small interfering RNA (siRNA) was provided by Tsingke Biotech Co. (Beijing, China). Following the screening, the siRNA sequence was obtained as follows:

*Sense* GCAAGCGGAUCCAGAUCUA.

*Anti-sense* UAGAUCUGGAUCCGCUUGC.

After achieving the optimal cell state and fusion degree, NRCMs cells were placed in 6-well plates and then subjected to transfection with 5 μl of siRNA (50 nM) using RNAiMAX, following the instructions provided in the kit. 24 h post-transfection, NRCMs were treated with ISO to induce myocardial hypertrophy along with XBW drugs. After an additional 24 h, the cells were collected for further treatment.

### Flow cytometry

The NRCMs cells were seeded in 6-well plates. After drug administration, the cells were cultured in fresh serum-free DMEM medium supplemented with 50 μM of 2-NBDG for 1 h. Subsequently, trypsin (0.25%) was used to detach and terminate the cells, which were then neutralized using a serum-containing medium. The resulting suspension was centrifuged, and the supernatant was discarded while retaining the cell pellet. After the cells were resuspended in PBS, a flow cytometer was utilized to analyze the fluorescence intensity. The excitation wavelength employed for this analysis was 488 nm.

### Statistical analysis

The data were presented as mean ± standard error (*Mean* ± *SEM*) and analyzed using GraphPad Prism 9.0. (San Diego, California, USA). *T-test* was used to compare two groups, and multiple groups were compared using *One-way* analysis of variance (*One-way ANOVA, Bonferroni* method). Statistical significance was determined when the *P*-value was less than 0.05.

## Results

### XBW effectively mitigates *ISO*-induced myocardial hypertrophy in NRCMs and modulates the expression of enzymes involved in myocardial fatty acid metabolism

**Fig. 1:**
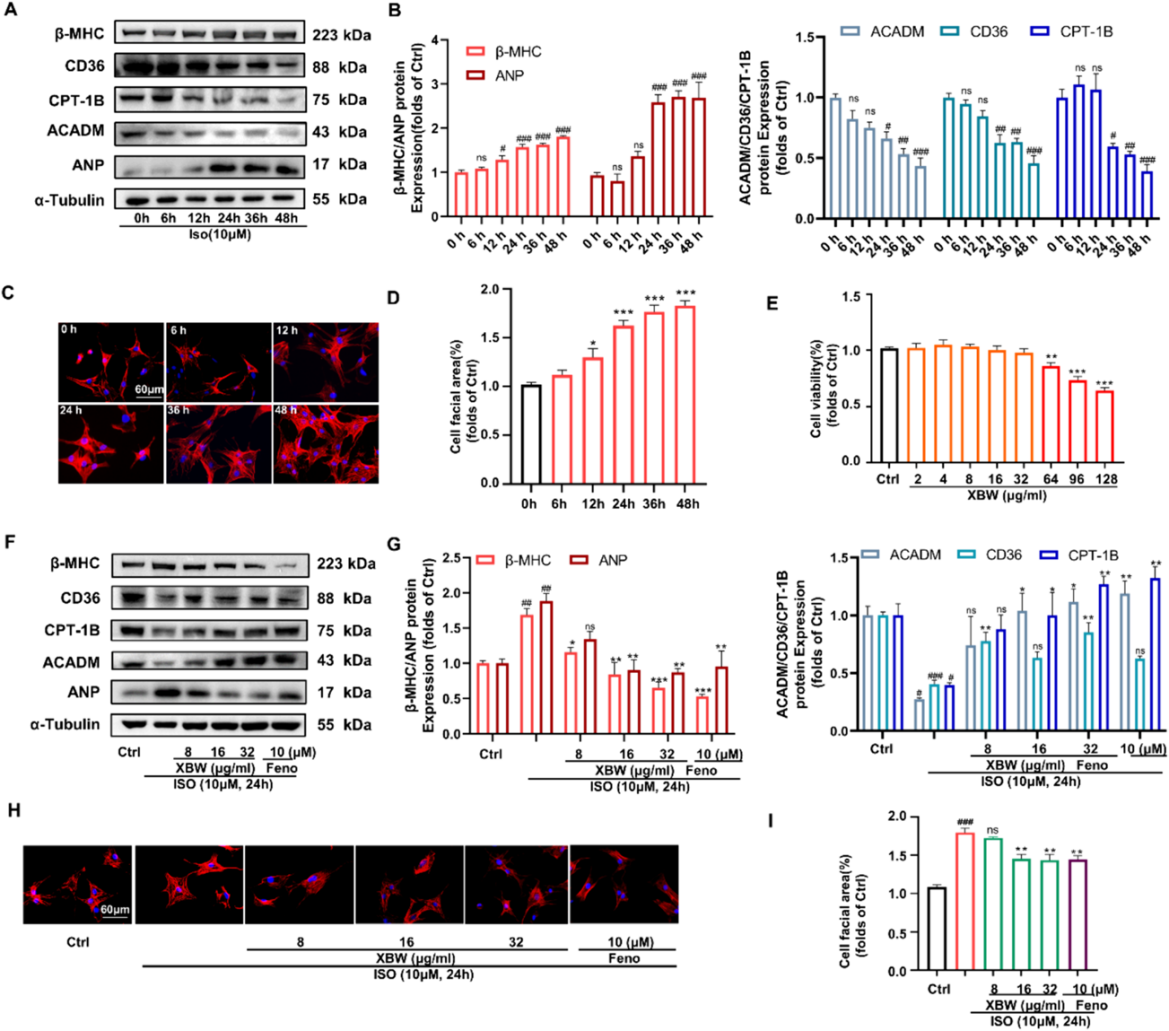
XBW effectively mitigates ISO-induced myocardial hypertrophy in NRCMs and modulates the expression of enzymes involved in myocardial fatty acid metabolism. **A**–**B** In NRCMS, Western Blot detected alterations in β-MHC and ANP protein levels, indicators of ISO-induced myocardial hypertrophy, as well as CD36, CPT-1B, and ACADM, markers of cardiac FA energy metabolism. **C**–**D** ISO-induced alterations in cell surface area of NRCMs within 48 h. The cell surface area was meticulously evaluated via Rhodamine-Phalloidin staining. Scale bar: 60 μm. **E** The viability of NRCMs was assessed using the MTT assay after incubation with various concentrations of XBW for 24 h. **F**–**G** After ISO stimulation and drug treatment, the WB analysis assessed the protein contents of β-MHC, ANP, ACADM, CD36, and CPT-1B. **H**–**I** The alterations in cell surface area following XBW administration were quantified. Scale bar: 60 μm. Compared with Ctrl group, ^###^*P* < 0.001, ^##^*P* < 0.01, ^#^*P* < 0.05, ns: no significance; compared with ISO-induced group, ^***^P < 0.001, ^**^P < 0.01, ^*^P < 0.05, ns: no significance

ISO is commonly utilized to establish HF models [23]. Therefore, we employed ISO to induce hypertrophy and energy metabolism disorder in NRCMs and to evaluate the impact of XBW on HF in vitro. NRCMs were treated with 10 μM ISO for various durations (0, 6, 12, 24, 36, and 48 h), and the levels of protein expression for cardiac hypertrophy markers (β-MHC, ANP) and cardiac energy metabolism markers (CD36, CPT-1B, ACADM) were assessed. The results revealed a time-dependent increase in β-MHC and ANP protein expression following ISO stimulation **(**Fig. 1A–B**)**. In contrast, the protein expression of myocardial energy metabolism indicators (CD36, CPT-1B, ACADM) was decreased as the duration of ISO stimulation was increased in a time-dependent manner **(**Fig. 1A–B**)**. Rhodamine-Phalloidin staining for cell surface area demonstrated a substantial enlargement of NRCMs after exposure to ISO stimulation after 24 h **(**Fig. 1C–D**)**. Based on the aforementioned experimental results **(**Fig. 1A–D**)**, we chose NRCMs that were stimulated with 10 μM ISO for 24 h to serve as an in vitro representation of HF. MTT assay results revealed that XBW exhibited no cytotoxicity towards NRCMs within a concentration range of 2–32 μg/mL **(**Fig. 1E). Subsequently, for our further in vitro experiments, intervention concentrations of 8, 16, and 32 μg/mL were chosen based on evaluating the drug’s efficacy. Furthermore, our study revealed that XBW exhibited a dose-dependent effect on diminishing the protein levels of hypertrophy markers β-MHC and ANP in our HF cell model induced by ISO at varying concentrations (8, 16, and 32 μg/mL). Moreover, XBW effectively mitigated the ISO-induced enlargement of the myocardial surface area. Besides, XBW notably enhanced the protein levels of myocardial energy metabolism markers CD36, CPT-1B, and ACADM (Fig. 1F-G). Meanwhile, XBW significantly ameliorates the abnormal surface area enlargement of NRCMs induced by ISO (Fig. 1H-I). The combined results show that XBW successfully reduced ISO-induced hypertrophy and controlled CD36, CPT-1B, and ACADM expression levels in NRCMs.

### XBW improves cardiac function in rats with heart failure induced by *ISO*

**Fig. 2:**
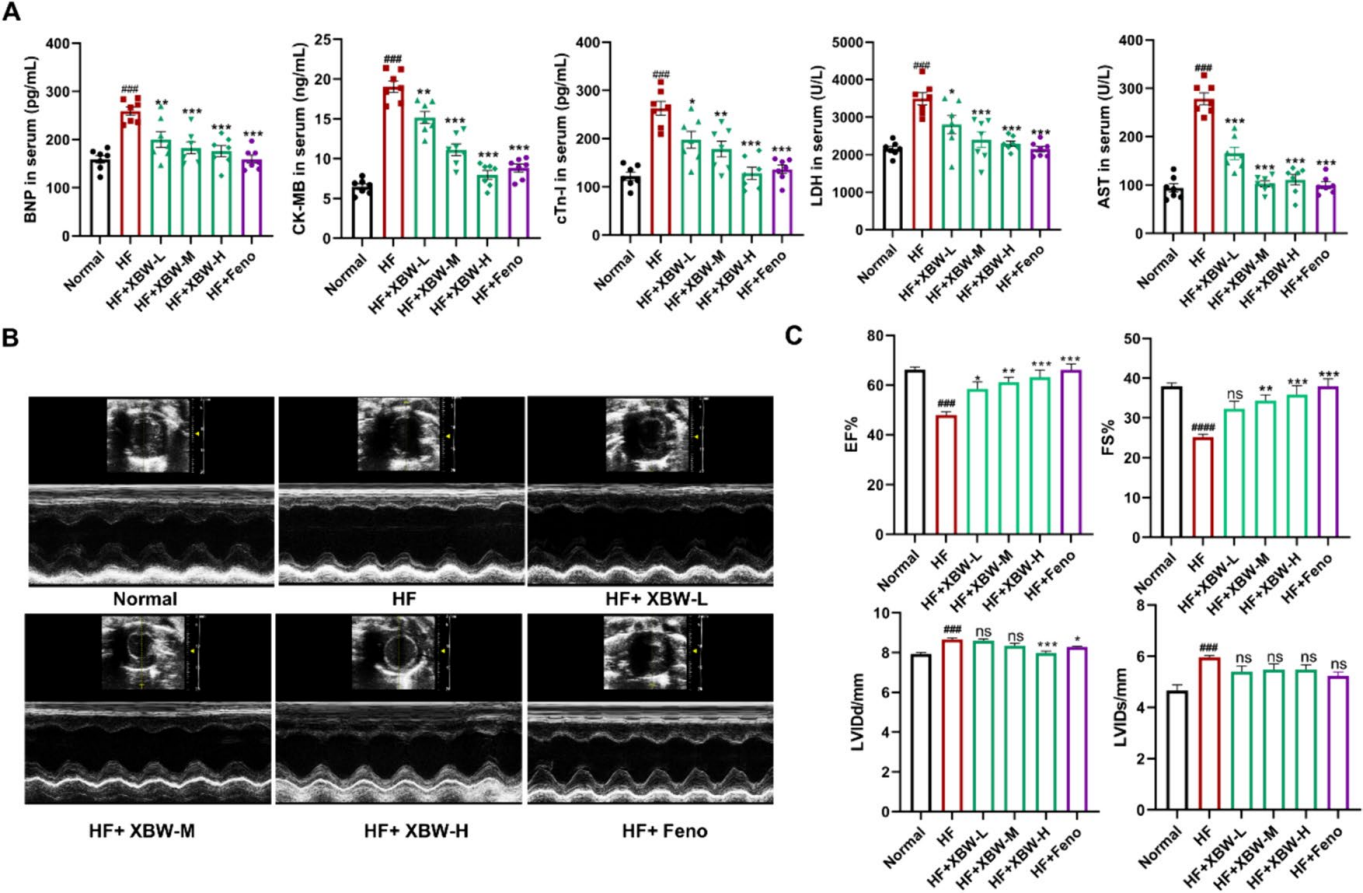
XBW improves cardiac function in rats with heart failure induced by ISO. **A** Myocardial injury markers, including BNP, CK-MB, cTn-I, LDH, and AST, were analyzed in the serum of rats from different groups. **B** Echocardiography M-mode images from the left ventricles of various experimental groups are presented as representative examples.** C** Cardiac function was evaluated by quantifying echocardiographic parameters such as EF%, FS%, LVIDd, and LVIDs. Compared with Normal group, ^###^*P* < 0.001, ^##^*P* < 0.01, ^#^*P* < 0.05; compared with HF group, ^***^P < 0.001, ^**^P < 0.01, ^*^P < 0.05, ns: no significance,* n* = 7. HF: ISO subcutaneous injection induced-HF group; HF + XBW-L: XBW treatment group (low dose: 60 mg/kg); HF + XBW-M: XBW treatment group (medium dose: 80 mg/kg); HF + XBW-H: XBW treatment group (high dose: 120 mg/kg); HF + Feno: fenofibrate treatment group (100 mg/kg)

Myocardial injury-related biomarkers in the blood, such as BNP, CK-MB, cTn-I, and LDH, are commonly employed for HF auxiliary evaluation and diagnosis. After XBW treatment, the five serum indicators of myocardial injury showed significant decreases **(**Fig. 2A**)**. The changes in the cardiac function of rats with heart failure were observed using echocardiography **(**Fig. 2B**)**. Meanwhile, the cardiac function of the experimental rats was assessed by quantifying parameters including LVEF, LVFS, LVIDd, and LVIDs [24]. During this investigation, we observed a notable decline in the LVEF and LVFS within the HF group, with mean percentages of 47.977% and 25.156%, respectively. In contrast, there was a notable rise in the LVIDd and LVIDs, measuring 8.675 mm and 5.958 mm, respectively (Fig. 2C). This suggests that ISO has the potential to induce HF, exacerbate cardiac abnormalities and hinder the ability of the heart to contract. Treatment with XBW or fenofibrate resulted in improved systolic function and cardiac pumping capacity, as indicated by LVEF values of 63.081% and LVFS values of 35.842% in the high-dose group of XBW. Additionally, XBW at a high dose decreased LVIDd values to 7.992 mm, with no significant change in LVIDs.

### XBW ameliorates structural abnormalities in myocardial tissue of heart failure rats induced by *ISO*

**Fig. 3:**
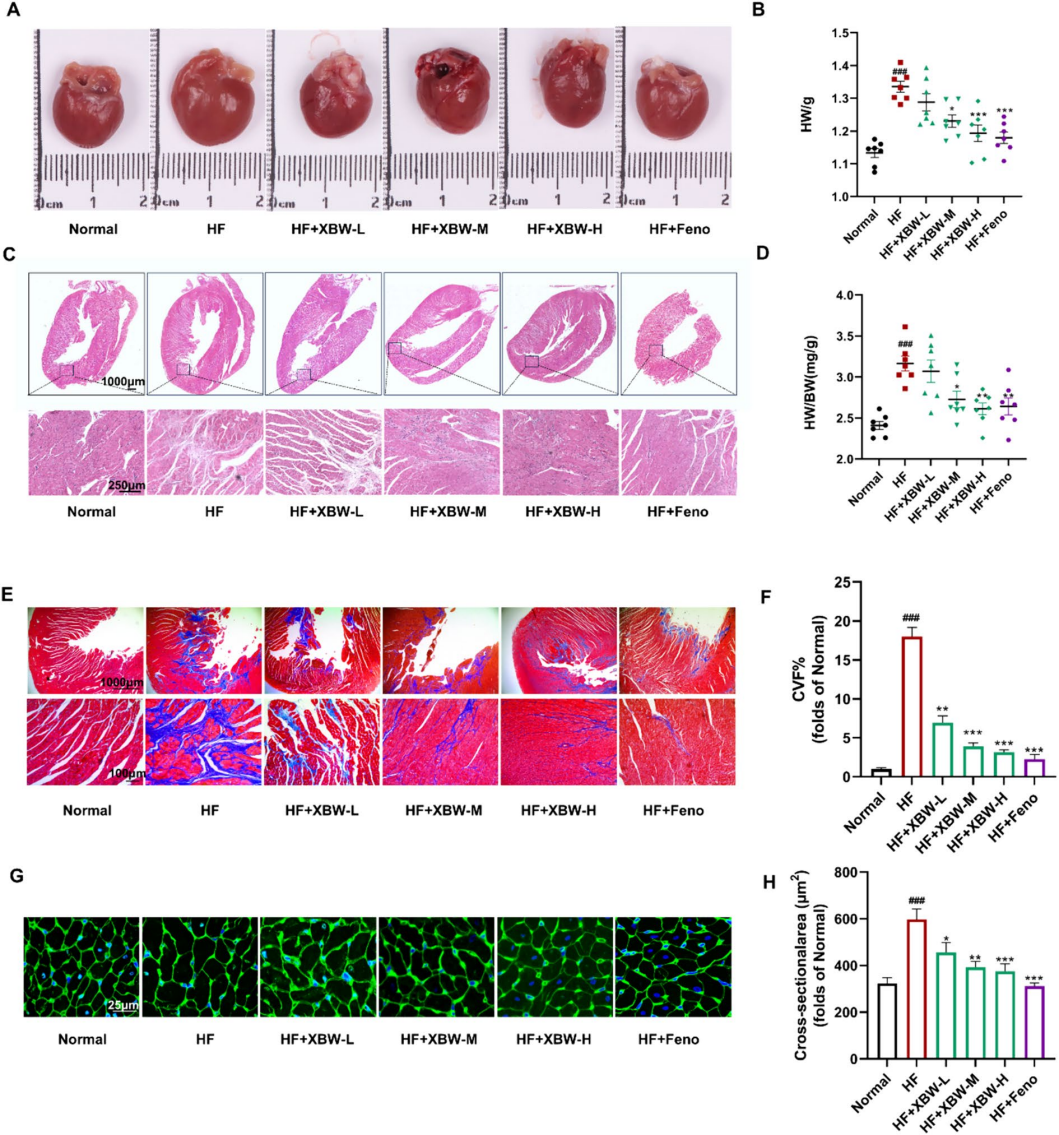
XBW ameliorates structural abnormalities in myocardial tissue of heart failure rats induced by ISO. **A** Intact cardiac morphological images of rats in various experimental groups. **B** Heart weights of rats in each group. **C** Representative HE-stained heart sections. Scale bar: 1000 μm. Scale bar: 250 μm. **D** Heart weight-to-body weight ratio of rats. **E** Masson-stained heart sections in each group. Scale bar: 100 μm. **F** Quantitative analysis of collagen volume in the left ventricles. **G** WGA-stained sections of representative rat hearts from each group. Scale bar: 25 μm. **H** Quantitative analysis of the cross-sectional area. Compared with Normal group, ^###^*P* < 0.001, ^##^*P* < 0.01, ^#^*P* < 0.05; compared with HF group, ^***^P < 0.001, ^**^P < 0.01, ^*^P < 0.05

To further confirm the protective effect of XBW on the heart, we conducted experiments and examined pathological sections of rats for validation. Compared to the control group, rats induced with ISO exhibited higher heart volume and a significantly increased heart weight/heart weight ratio. Conversely, the XBW treatment and positive drug groups demonstrated enhanced heart morphology and a notable decrease in the heart weight/heart weight ratio. Notably, the high-dose XBW group exhibited a similar performance to the positive drug group (Fig. 3A-B, D). Similarly, HE and Masson staining were used to observe cardiomyocyte arrangement tissue structure and evaluate myocardial collagen formation levels. In the HE staining sections, HF rats exhibited a disordered arrangement of cardiomyocytes, an increased area of cardiomyocytes, and a high degree of collagenization. However, this situation was improved following XBW treatment compared to HF (Fig. 3C). The results from the Masson staining sections also confirmed that XBW reduced collagen levels in cardiac tissues caused by HF (Fig. 3E–F). Additionally, WGA-stained sections were prepared to investigate the cellular morphology in cardiac tissue further. Based on these observations, it was noted that cardiomyocytes in the HF group showed an irregular arrangement along with an increased cross-sectional area. In contrast, myocardial tissue from rats treated with XBW showed an improvement with a regular arrangement and a significant reduction in the cross-sectional area of cells (Fig. 3G–H). The results indicate that XBW can potentially mitigate pathological alterations in the heart and enhance myocardial tissue structure in rats with HF.

### XBW exhibits cardioprotective effects by mitigating the pathological alterations associated with HF and modulating fatty acid metabolism in *ISO*-induced HF rats

**Fig. 4:**
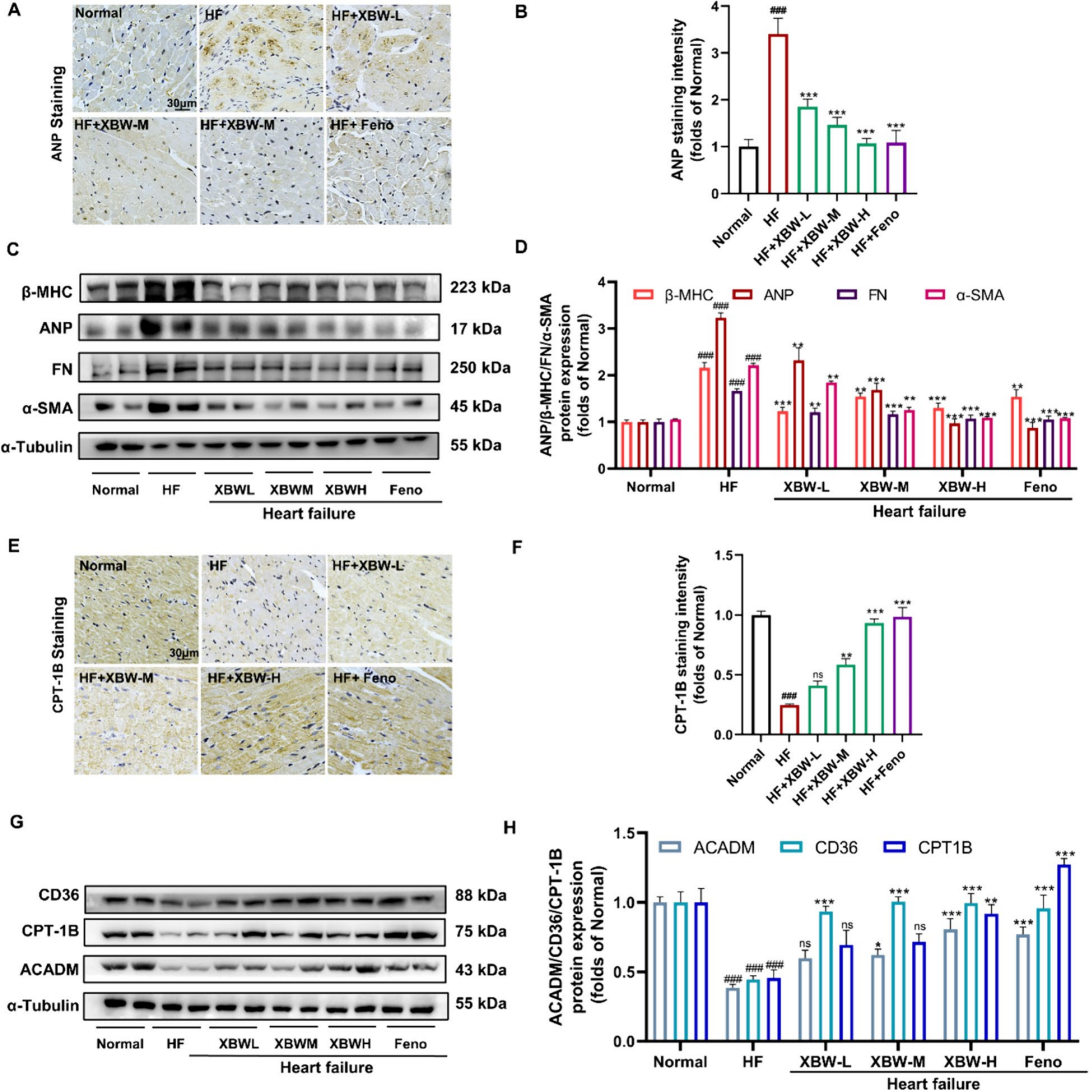
XBW exhibits cardioprotective effects by mitigating the pathological alterations associated with HF and modulating fatty acid metabolism in ISO-induced HF rats. **A**–**B** Immunohistochemical staining was performed to assess the expression of ANP protein. Scale bar: 30 μm. **C–D** WB was employed to determine the protein levels of markers associated with hypertrophy and fibrosis in cardiac tissue. **E**–**F** Immunohistochemistry was utilized to examine the expression of CPT-1B protein. Scale bar: 30 μm. **G**–**H** The ACADM, CD36, and CPT-1B protein levels were assessed using WB analysis. Compared with Normal group, ^###^*P* < 0.001, ^##^*P* < 0.01, #*P* < 0.05; compared with HF group, ^***^P < 0.001, ^**^P < 0.01, ^*^P < 0.05, ns: no significance

Subsequently, we investigated the effects of XBW on cardiac hypertrophy factors (ANP, β-MHC), cardiac fibrosis factors (FN, α-SMA), and cardiac fatty acid metabolism factors (CD36, CPT-1B, ACADM) in rats with ISO-induced HF. Firstly, immunohistochemistry and Western blotting experiments revealed that the protein levels of cardiac hypertrophy markers (β-MHC, ANP) and fibrosis markers (FN, α-SMA) were significantly increased in the left ventricle of rats in the HF group compared with the Normal group. However, treatment with XBW or Feno resulted in substantially lower protein expression levels of cardiac hypertrophy and fibrosis markers in the left ventricular myocardium compared with the HF group, consistent with in vitro results (Fig. 4A–D). In addition, our research showed that in the left ventricle of ISO-induced HF rats, the protein levels of related fatty acid metabolism factors (CD36, CPT-1B, ACADM) were significantly decreased, which was significantly improved after treatment with XBW or Feno (Fig. 4E–H). These findings indicate that XBW ameliorated ISO-induced cardiac hypertrophy and dysregulation of fatty acid metabolism.

### XBW significantly enhances cardiac energy levels and alleviates mitochondrial functional damage in *ISO*-induced NRCMs

**Fig. 5:**
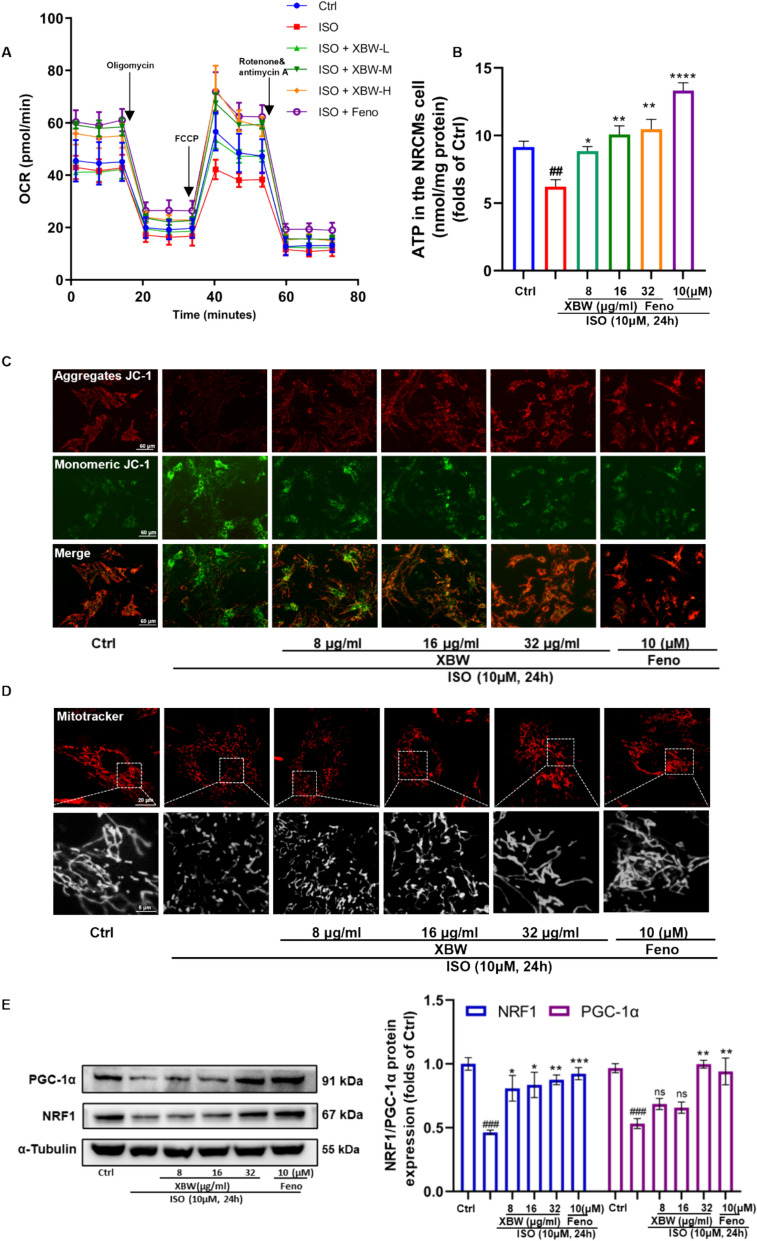
XBW significantly enhances cardiac energy levels and alleviates mitochondrial functional damage in ISO-induced NRCMs.** A** The XF Cell Mito Stress Test was employed to assess alterations in OCR levels in ISO-induced NRCMs. **B** The ATP content levels in NRCMs following drug intervention. **C** The mitochondrial membrane potential of NRCMs was investigated using the JC-1 Fluorescent probe. Scale bar: 60 μm. **D** The mitochondrial morphology of NRCMs was visualized via the Mito-Tracker Red CMXRos probe. Scale bar: 60 μm or 20 μm. **E** WB analysis was employed to assess the alterations in the levels of PGC-1α and NRF1, which are associated with mitochondrial biogenesis. Independent experiments were performed at least three times with similar results. Compared with Ctrl group, ^###^*P* < 0.001, ^##^*P* < 0.01, ^#^*P* < 0.05; compared with ISO-induced group, ^***^P < 0.001, ^**^P < 0.01, ^*^P < 0.05, ns: no significance

Mitochondria are the central hub for cellular energy metabolism, supplying abundant energy to facilitate various physiological functions in cells. HF can potentially disrupt myocardial energy supply and impair energy metabolism [25]. In cardiomyocytes, HF mainly triggers mitochondrial damage, encompassing morphological changes and functional defects. In ISO-treated NRCMs, XBW significantly ameliorated the decrease in OCR levels, alleviated mitochondrial dysfunction and improved ATP generation **(**Fig. 5A–B**)**. Meanwhile, XBW treatment restored and enhanced the reduced mitochondrial membrane potential induced by ISO **(**Fig. 5C**)**. Furthermore, XBW administration alleviated the alterations in mitochondrial morphology observed in NRCMs **(**Fig. 5D**)**. In addition, XBW exhibited a dose-dependent effect on enhancing the protein expression levels of particular indicators strongly associated with the functioning of mitochondria, which included PGC-1α and NRF1 **(**Fig. 5E**)**. Collectively, XBW significantly increases myocardial energy levels and mitigates ISO-induced mitochondrial dysfunction.

### XBW may modulate the AMPK/PPARα signalling *axis* through SGLT1 inhibition, thereby enhancing myocardial energy supply

**Fig. 6:**
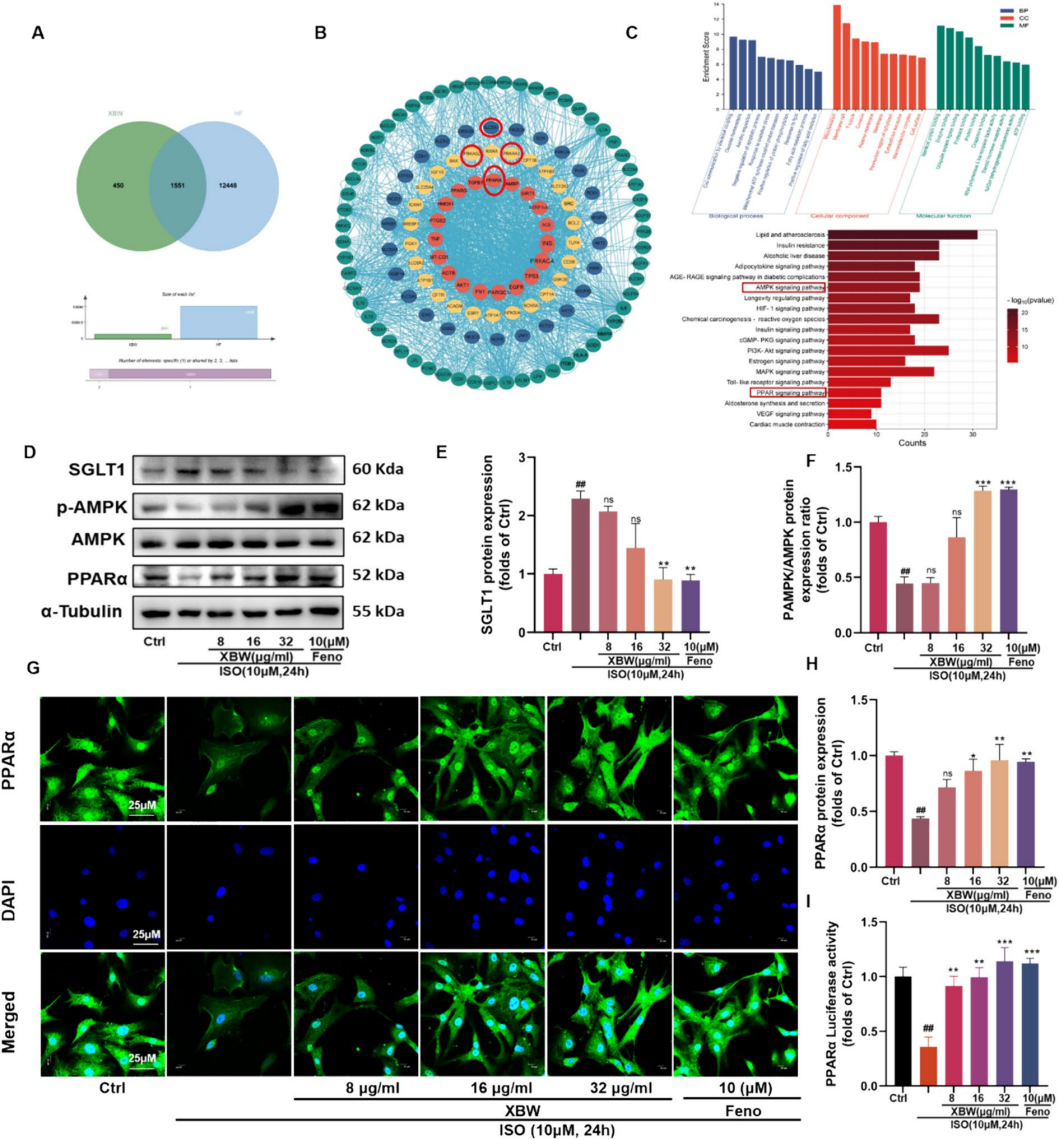
XBW may modulate the AMPK/PPARα signalling axis through SGLT1 inhibition, thereby enhancing myocardial energy supply.** A** Venn diagram of the intersection between XBW and HF. **B** PPI network interaction diagram. **C** GO and KEGG analyses of dominant targets. **D**–**F, H** WB was employed to assess the changes of the SGLT1/AMPK/PPARα axis following XBW treatment. **G** Immunofluorescence was utilized to detect the protein expression of PPARα in NRCMs. Scale bar: 25 μm. **I** PPARα luciferase activity was quantified using a dual luciferase reporter gene assay. Independent experiments were performed at least three times with similar results. Compared with Ctrl group, ^###^*P* < 0.001, ^##^*P* < 0.01, ^#^*P* < 0.05; compared with ISO-induced group, ^***^ *P* < 0.001, ^**^P < 0.01, ^*^P < 0.05, ns: no significance

**Fig.7:**
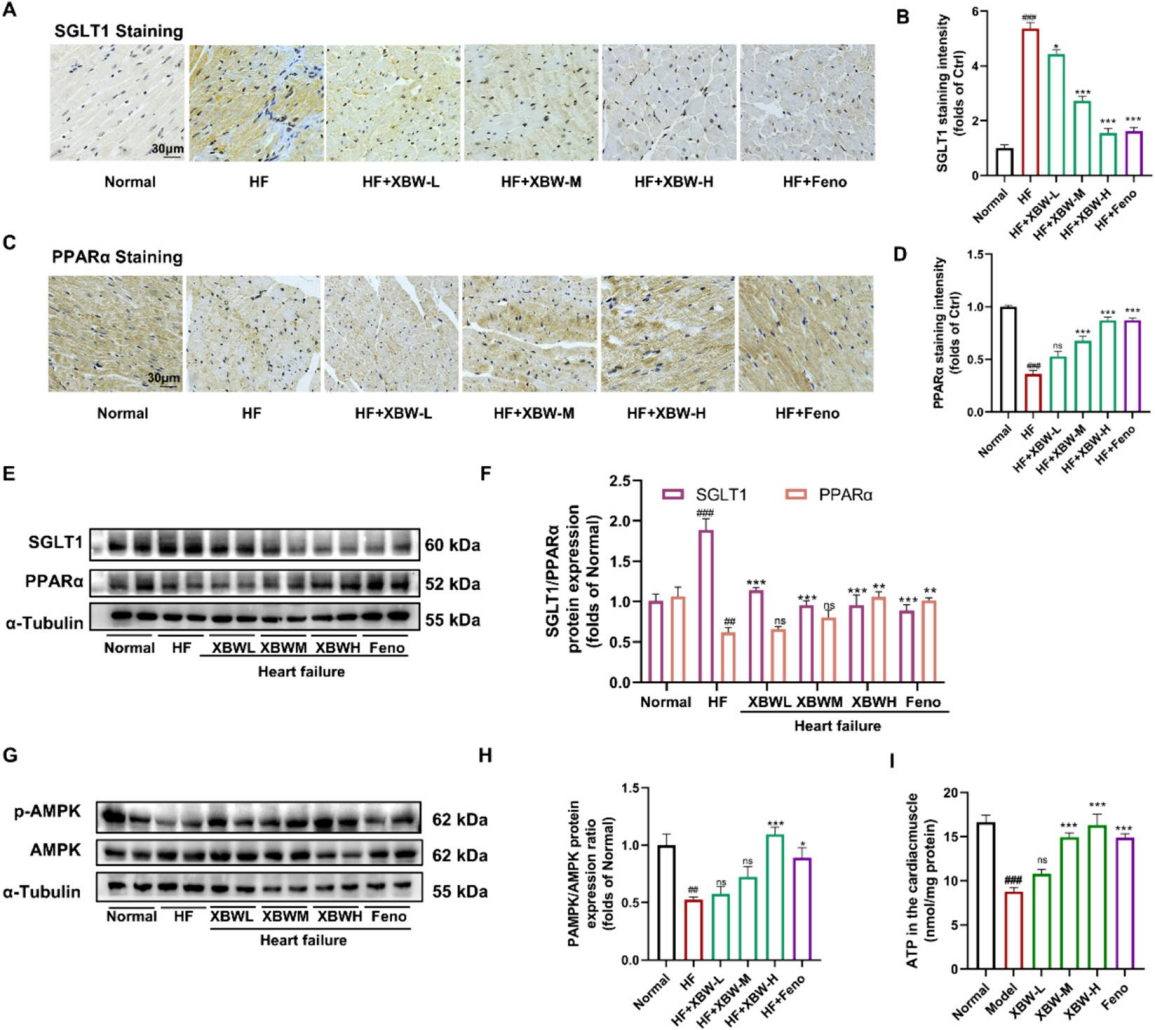
XBW may modulate the AMPK/PPARα signalling axis through SGLT1 inhibition, thereby enhancing myocardial energy supply. **A**–**B** Immunohistochemical staining was employed to assess the protein expression of SGLT1 in myocardial tissue from each experimental group. Scale bar: 30 μm. **C–D** PPARα protein levels were evaluated using immunohistochemistry. Scale bar: 30 μm. **E**–**H** WB analysis was utilized to determine the expression levels of SGLT1 and PPARα proteins, as well as the ratio of p-AMPK to AMPK protein expression. **I** ATP content in myocardial tissue from each experimental group was analyzed. Compared with Normal group, ^###^*P* < 0.001, ^##^*P* < 0.01, ^#^*P* < 0.05; compared with HF group, ^***^P < 0.001, ^**^P < 0.01, ^*^P < 0.05, ns: no significance

To further examine the fundamental processes of XBW in improving the supply of energy to the heart and the metabolism of energy in the myocardium for the treatment of HF, we utilized network pharmacology to investigate its possible targets. We initially identified potential targets for XBW to exert cardioprotective effects by extracting data from the ETCM and Herb databases. Simultaneously, utilizing DisGeNET and Genecards, potential disease targets related to “Heart failure,” “Chronic heart failure,” “Cardiomegaly,” and “Myocardial hypertrophy” were acquired. Through the intersection of these two sets, we discovered the potential targets that overlap, and we represented this visually using a Venn diagram **(**Fig. 6A**)**. In order to assess the priority and significance of these potential targets, we utilized Genecards and STRING 11.0 database for screening out the core target set for further analysis, followed by conducting network topology analysis on the targets to construct an interactive network. Based on the analysis and screening principle, we selected a set of 130 critical targets with eigenvalues (BC) above the median value as our focal points. Subsequently, the PPI network was constructed based on these key target sets using Cytoscape software **(**Fig. 6B**)**. Additionally, we conducted GO and KEGG analyses using the David and Metascape databases, respectively, based on the dominant potential target set **(**Fig. 6C**).** WB results revealed a significant upregulation in the expression of SGLT1 in the model group compared to the normal group, while the phosphorylation level of AMPK and protein content of PPARα were markedly reduced**.** Following intervention with XBW or fenofibrate, there was a significant down-regulation of SGLT1. Moreover, the activation of the AMPK/PPARα signalling axis was observed by XBW since the phosphorylation of AMPK was elevated, and PPARα expression was increased (Fig. 6D–F, H and Fig. 7E–H), consistent with the immunohistochemical results in heart tissue **(**Fig. 7A–D**)**. The IF analysis revealed a significant decrease in PPARα induced by ISO. However, treatment with XBW significantly increased PPARα protein content within cells and facilitated its translocation into the nucleus **(**Fig. 6G**)**. Additionally, XBW increased the transcriptional activity mediated by PPARα in ISO-induced NRCMs in a dose-dependent manner **(**Fig. 6I**)**. Furthermore, the myocardial ATP levels revealed a substantial enhancement following XBW treatment, compared to the model group** (**Fig. 7I**)**.

### SGLT1 silencing enhances the potential of XBW in attenuating myocardial pathologic changes and enhancing myocardial fatty acid energy metabolism

**Fig. 8:**
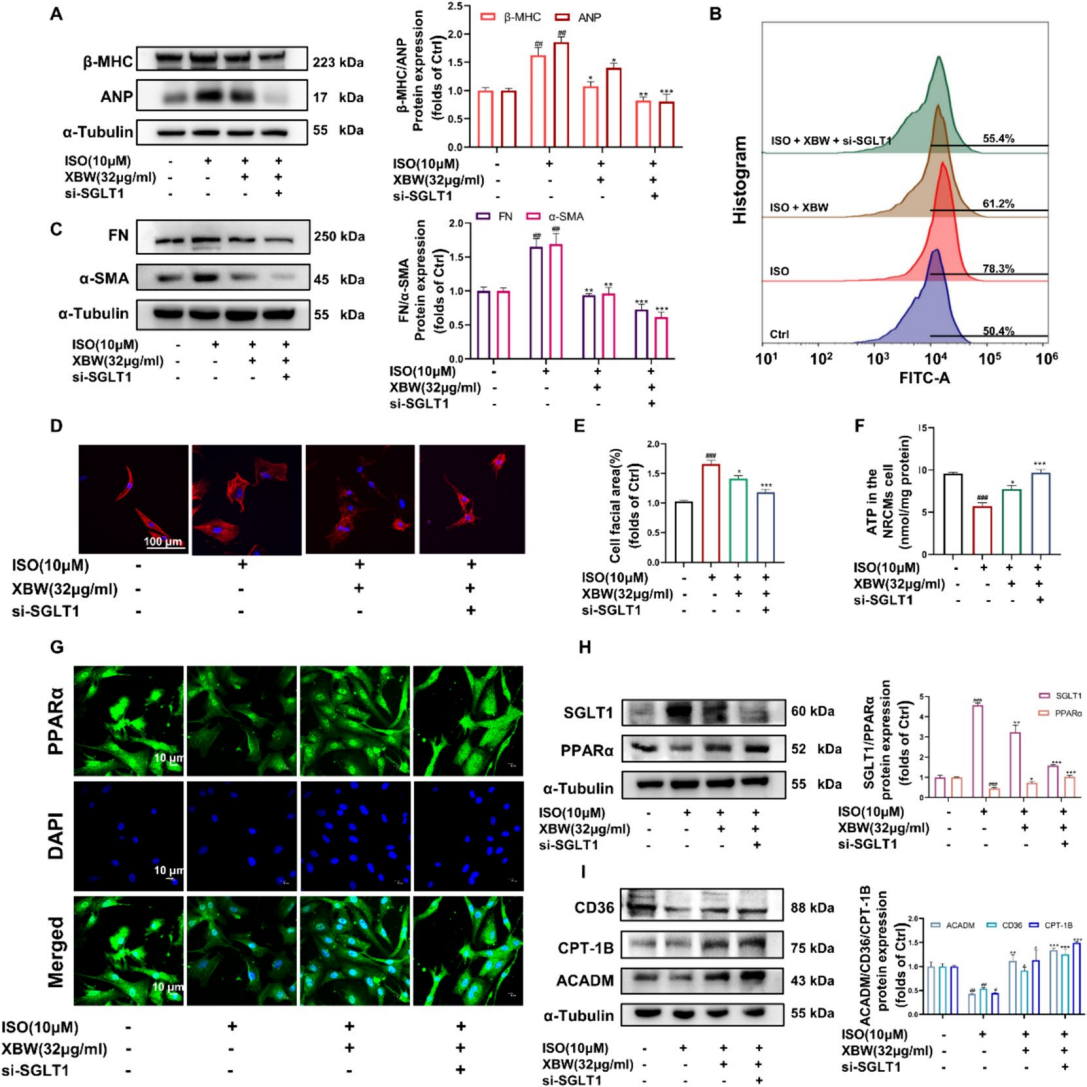
SGLT1 silencing enhances the potential of XBW in attenuating myocardial pathologic changes and enhancing myocardial fatty acid energy metabolism. **A** The effects of XBW on alterations in the protein levels of myocardial hypertrophy markers β-MHC and ANP after SGLT1 silence. **B** The effect of XBW on intracellular glucose transport after silencing SGLT1 was measured by flow cytometry. **C** The protein levels of FN and α-SMA, which serve as biomarkers for myocardial fibrosis, were assessed via WB analysis. **D**–**E** The effect of XBW on changes in cell surface area visualized through Rhodamine-Phalloidin staining following SGLT1 interference. Scale bar: 100 μm. **F** NRCMs’ ATP content level. **G** Immunofluorescence was employed to examine PPARα expression after silencing SGLT1. Scale bar: 10 μm. **H**–**I** WB analysis was conducted to evaluate the protein expression of SGLT1, PPARα, and myocardial fatty acid energy metabolism indicators CD36, CPT-1B, and ACADM. Independent experiments were performed at least three times with similar results. Compared with Ctrl group, ^###^*P* < 0.001, ^##^*P* < 0.01, ^#^*P* < 0.05; compared with ISO-induced group, ^***^P < 0.001, ^**^P < 0.01, ^*^P < 0.05

To further investigate the potential impact of XBW by targeting SGLT1 on HF, we employed small interfering RNA to knock down SGLT1 in NRCMs. WB analysis showed that SGLT1 silencing enhanced the effect of XBW on reducing myocardial hypertrophy and fibrosis **(**Fig. 8A, C**)**. Furthermore, silencing of SGLT1 further enhanced XBW’s ability to reduce the ISO-induced increase in cell surface area **(**Fig. 8D–E**).** Flow cytometry was utilized to evaluate the impact of XBW on glucose transport. Compared to the control group, the model group exhibited disrupted myocardial energy metabolism, demonstrated by a significant increase in fluorescence intensity and intracellular 2-NBDG content, indicating elevated intracellular glucose levels and an imbalanced glucose-lipid metabolism. Administration of XBW led to decreased fluorescence intensity compared to the model group, restoring the balance of glucose-lipid metabolism. After the silence of SGLT1, fluorescence intensity was further reduced relative to the XBW-treated group (Fig. 8B). Additionally, the ATP assay results demonstrated that the potentiation effect of XBW was further augmented by SGLT1 deficiency (Fig. 8F). To confirm further the impact of XBW on PPARα activation following SGLT1 knockdown, IF was employed to examine the cellular distribution of PPARα. IF results demonstrated that SGLT1 silencing further enhanced the nuclear distribution of PPARα under XBW administration, thereby significantly augmenting XBW’s regulation of proteins associated with myocardial energy metabolism **(**Fig. 8G–I**)**. In conclusion, SGLT1 knockdown enhances the potential of XBW to reduce myocardial hypertrophy and improve myocardial fatty acid energy metabolism.

### SGLT1 overexpression blocked the effectiveness of XBW in treating myocardial pathologic changes and improving cardiac fatty acid energy metabolism

**Fig. 9:**
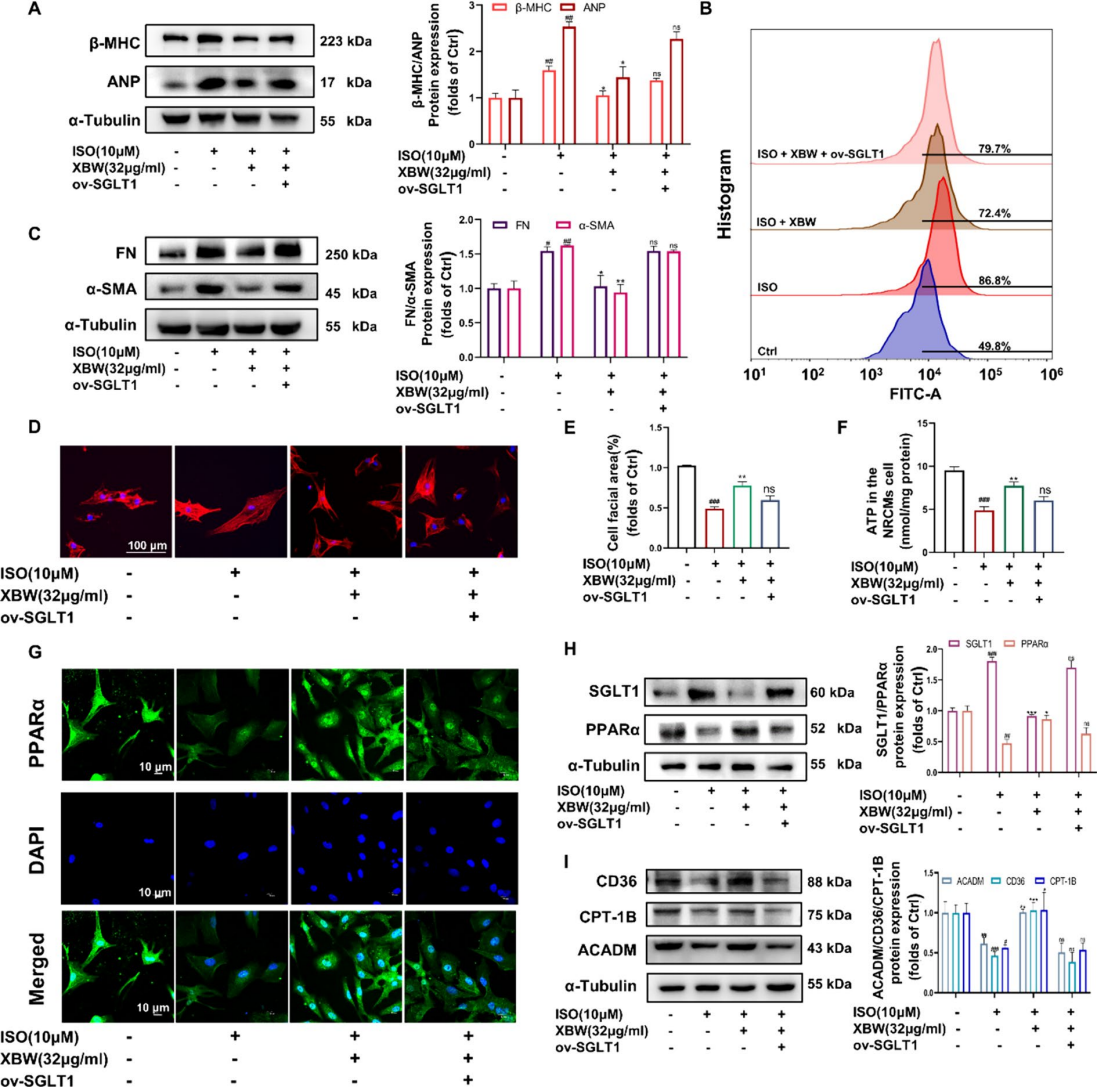
SGLT1 overexpression blocked the effectiveness of XBW in treating myocardial pathologic changes and improving cardiac fatty acid energy metabolism** A** The protein levels of β-MHC and ANP under SGLT1 overexpression. **B** The effect of XBW on intracellular glucose transport subjected to SGLT1 overexpression. **C** The protein levels of FN and α-SMA were assessed via WB analysis. **D**–**E** Cell surface area was determined via Rhodamine-Phalloidin staining. Scale bar: 100 μm **F** Cellular ATP content levels were assessed. **G** IF analysis disclosed alterations in PPARα protein expression. **H**–**I** The protein contents of SGLT1, PPARα, CD36, CPT-1B, and ACADM were assessed using WB analysis to investigate the impact of SGLT1 overexpression across different experimental groups. Independent experiments were performed at least three times with similar results. Compared with Ctrl group, ^###^*P* < 0.001, ^##^*P* < 0.01, ^#^*P* < 0.05; compared with ISO-induced group, ^***^P < 0.001, ^**^P < 0.01, ^*^P < 0.05, ns: no significance

Furthermore, we investigate the impact of XBW against HF under overexpression of SGLT1 in NRCMs. WB analysis showed a significant attenuation of XBW’s inhibitory effect on β-MHC, ANP, FN, and α-SMA protein expression when SGLT1 was overexpressed **(**Fig. 9A, C**)**. Moreover, overexpression of SGLT1 reversed the ability of XBW to ameliorate the ISO-induced increase in the cell surface area of NRCMs **(**Fig. 9D–E**)**. The inhibitory effect of XBW on glucose uptake was found to be significantly attenuated upon overexpression of SGLT1, as evidenced by the flow cytometry results **(**Fig. 9B**)**. The results of the ATP content determination experiment also indicated that the overexpression of SGLT1 significantly attenuated the impact of XBW on enhancing intracellular ATP levels **(**Fig. 9F**)**. IF study, demonstrated a significant reduction in PPARα expression levels to a similar extent as that seen in the model group when SGLT1 was overexpressed **(**Fig. 9G**)**. In addition, Western Blot analysis revealed that the ameliorative effect of XBW on PPARα and its downstream protein levels was reversed upon overexpression of SGLT1 **(**Fig. 9H–I**)**. As a result, the capacity of XBW to alleviate cardiac hypertrophy and enhance cardiac fatty acid energy metabolism was reversed following SGLT1 overexpression. These results confirm that suppression of SGLT1 is involved in XBW’s cardo-protective effects.

## Discussion

In our research, ISO was used to establish in vivo and in vitro models of HF due to its non-selective agonistic activity at the β-adrenergic receptor, which mimics the pathogenesis of cardiovascular diseases as a result of abnormal sympathetic activation [26, 27]. As a result, ISO is often used in HF models in cardiovascular research. Extensive research data have consistently shown that 10 μM ISO effectively induces myocardial hypertrophy [28], myocardial fibrosis [29], cardiomyocyte necrosis [30], and disturbances in cardiac energy metabolism [31] within a 24 h stimulation period, which is in line with our observations.

XBW has been used in clinical settings for more than 30 years and has significant efficacy in treating HF [32]. Herbs within the XBW formula can regulate the nervous system, RAS system, mitophagy, and water-salt balance during HF development processes while reducing pathological changes such as cardiac hypertrophy or fibrosis, ultimately improving myocardial remodelling [33–35]. Regulation of myocardial energy metabolism is a novel therapeutic strategy for treating HF. This aims to rebalance the energy supply in the failing heart [36] and enhance overall cardiac energy levels, effectively arresting HF progression and treating the condition. Therefore, improving myocardial energy metabolism and enhancing myocardial fatty acid utilization are practical approaches for treating HF [37]. During our investigation, we found that XBW greatly enhances the expression of enzymes related to the oxidation of fatty acids, consequently facilitating the utilization of fatty acids while ameliorating mitochondrial dysfunction. In addition, XBW effectively alleviates pathological changes like myocardial hypertrophy and fibrosis while enhancing overall energy supply levels. The discovery of these results is essential for preserving the energy levels of cardiomyocytes in cases of HF.

Through network pharmacology analysis, we identified that XBW primarily targets the AMPK/PPARα pathway. Several research studies have emphasized the regulatory function of this signalling pathway in regulating the metabolism of fatty acids and enhancing insulin resistance, HF, and non-alcoholic fatty liver disease [38–40]. The AMPK/PPARα axis undeniably has a vital function in governing the energy metabolism of myocardial fatty acids. AMPK can be activated by regulating the AMP/ATP ratio, leading to phosphorylation of threonine 172 on the AMPKα subunit [41]. That allows cytoplasmic AMPK to sense intracellular energy status and activate when it is insufficient, thereby increasing ATP content and enhancing the overall cellular energy supply [42]. PPAR belongs to the ligand-activated nuclear transcription factor superfamily and has three isoforms: PPARα, β, and γ, all expressed in cardiac tissue. Among these isoforms, PPARα exhibits the closest association with FA metabolism in cardiomyocytes [12]. Moreover, PPARα positively modulates fatty acid oxidation while negatively regulating glucose utilization in cardiomyocytes. Downstream gene regulation enhances mitochondrial oxidation to maintain a balanced state of myocardial energy metabolism [43]. In pathological conditions such as HF, there is downregulation of PPARα protein expression accompanied by increased glucose uptake and anaerobic glycolysis, along with reduced oxidative utilization of FA [44]. Previous research has shown that it effectively improves pathological alterations in a mouse model of HF induced by ISO and reduces damage associated with HF [45]. Our research showcased that XBW effectively triggers the activation of AMPK by enhancing the phosphorylation level of AMPK both in vitro and in vivo. XBW may cause an increase in the AMP/ATP ratio, which could be responsible for the subsequent activation of AMPK, leading to a significant enhancement in the transcriptional activity, content expression, and nuclear translocation of PPARα. The expression levels of relevant downstream target genes that are relevant to fatty acid metabolism were also markedly elevated. Simultaneously, activation of the XBW-induced AMPK/PPARα axis not only improves fatty acid utilization efficiency but also inhibits mitochondrial dysfunction. Under conditions of HF, these impacts are essential for sustaining the energy provision of cardiomyocytes.

SGLT1 is a Na^+^-glucose transporter integrated into the cell membrane’s lipid bilayer [46]. Unlike SGLT2, which is absent in normal or pathological cardiac tissues, studies pointed out that the mRNA transcription and protein expression levels of SGLT1 are significantly increased in the left ventricular tissues of patients with advanced HF and ischemia [47, 48]. Overexpression of SGLT1 induces reversible cardiac hypertrophy, fibrosis, and left ventricular dysfunction in mice [49]. Moreover, studies have also shown that canagliflozin, a dual inhibitor targeting SGLT1/2, effectively raises the AMP/ATP ratio by inhibiting SGLT1, subsequently activating AMPK phosphorylation, leading to the relief of cardiac injury caused by oxidative stress [50]. A previous report suggests that SGLT1 activates AMPK to regulate the AMP/ATP ratio [51], thereby potentially triggering the AMPK/PPARα signalling axis.

In our research, we initially conducted target mining using network pharmacology, revealing that the potential dominant targets of XBW were primarily associated with fatty acid energy metabolism, mitochondrial function, and AMPK/PPARα pathways. Notably, our investigation identified a strong correlation between SGLT1 protein and activation of the AMPK/PPARα axis. Subsequently, we found that the SGLT1 content level was significantly lower after XBW treatment than that of the model group through in vivo and in vitro experiments. After the silence of SGLT1, XBW significantly enhanced the activation of the AMPK/PPARα signalling axis, thereby improving cardiac hypertrophy and fibrosis, reducing glucose influx, promoting fatty acid energy metabolism, and increasing ATP supply. However, after overexpression of SGLT1, the protective effect of XBW on cardiomyocytes in an HF state was reversed. The results suggest that the AMPK/PPARα signalling axis and its subsequent impacts are significantly regulated by SGLT1, which is also essential for XBW’s cardioprotective properties.

Although numerous clinical studies have reported the efficacy of XBW in treating HF, there is a lack of systematic research into the mechanisms by which XBW improves myocardial energy metabolism. In view of this, the present study aims to investigate whether XBW can modulate myocardial fatty acid energy metabolism through a specific mechanism, thereby impeding the pathological progression of HF. This landmark study of XBW seeks to improve FA metabolism as a potential strategy to ameliorate HF.

As mentioned above, disturbances in cardiac energy metabolism serve as crucial indicators of the progression of HF, which is characterised by impaired FA metabolism and reduced FAO levels. Therefore, correcting the pathological changes in cardiac energy metabolism during the early stages of HF disease progression represents an effective therapeutic strategy for the management of HF [52]. Despite some studies suggesting that increased FAO may be detrimental to HF, such as the beneficial protective effect of the FAO inhibitor trimetazidine on failing hearts [53], there is also considerable evidence that improving FA metabolism may improve HF. For example, an increase in cardiac FAO levels by knockout of acetyl-CoA carboxylase 2 significantly improves myocardial hypertrophy [54]. In addition, L-carnitine, an FAO promoter, can improve cardiac FA metabolism by facilitating mitochondrial transport of FAs, and it has shown promising therapeutic effects in HF and clinical use [55]. Furthermore, Feno can activate the PPARα/Sirt1/PGC-1α signalling axis to increase gene expression related to FA metabolism in cardiac tissue and effectively inhibit the progression of HF [56]. In our research, we investigated the effects of XBW on FA metabolism in ISO-induced HF both in vivo and in vitro, elucidating its potential to ameliorate HF symptoms by improving FA metabolism and providing empirical support for this perspective. In addition, the pharmacological properties of XBW, which encompass multiple components, targets and pathways, not only significantly increase FAO but also improve myocardial oxygen delivery through multiple mechanisms, thereby counteracting the drawbacks associated with increased FAO leading to increased myocardial oxygen consumption. For example, Yangjinhua in XBW can improve blood flow velocity and increase oxygen delivery [57].

Moreover, our findings validate the efficacy of XBW in enhancing myocardial FA energy metabolism via the SGLT1/AMPK/PPARα signalling axis, thereby augmenting energy supply to cardiac tissue and ultimately impeding HF progression during the early to mid-stages, thus exerting an anti-HF effect [58]. Considering recent research reports alongside our study, timely improvement of myocardial fatty acid energy metabolism and failing heart’s energy supply holds significant therapeutic potential for HF treatment in its initial to intermediate phases. Furthermore, targeting the regulation of the SGLT1/AMPK/PPARα signalling axis to enhance myocardial energy metabolism also presents a novel perspective for future strategies in HF management.

Nevertheless, there are a few limitations in this study: (1) The inclusion of SGLT1 knockout mice can provide more insights into the correlation between SGLT1 and XBW treatment for HF; (2) Diverse modelling techniques can be utilized to perform in vivo and in vitro experiments that more accurately mimic the HF disease model and investigate its therapeutic mechanism.

## Conclusions

This research indicates that XBW suppresses SGLT1, followed by activation of the AMPK/PPARα signalling pathway, improving myocardial fatty acid energy metabolism and finally ameliorating myocardial hypertrophy and fibrosis. These findings provide novel insight into the understanding of XBW in treating HF, presenting a novel objective rationale for the clinical application of XBW in HF.

### Supplementary Information

Supplementary material 1.

## Data Availability

The datasets used and/or analysed during the current study are available from the corresponding author upon reasonable request.

## Abbreviations

ACADM: Medium-chain acyl-CoA dehydrogenase
AMP: Adenosine monophosphate
AMPK: Adenosine 5 ‘-monophosphate (AMP)-activated protein kinase
ANP: Atrial natriuretic factor
AST: Aspartate transaminase
ATP: Adenosine triphosphate
BNP: Brain natriuretic peptide
BW: Body weight
CD36: Cluster of differentiation 36
CK-MB: Creatine kinase MB isoenzyme
CMC-Na: Sodium carboxymethyl cellulose
CPT-1B: Carnitine palmitoyltransferase-1B
c-TnI: Cardiac troponin I
FFA: Free fatty acids
FN: Fibronectin
HDL-C: Cholesterol of high-density lipoprotein
HE: Hematoxylin–eosin
HW: Heart weight
ISO: Isoproterenol
LDH: Lactate dehydrogenase
LDL-C: Low-density lipoprotein
LVEF: Left ventricular ejection fraction
LVFS: Left ventricular fractional shortening
LVIDd: Left ventricular end-diastolic diameter
LVIDs: Left ventricular end-systolic diameter
MTT: Methyl thiazolytetrazolium
PPI: Protein–protein interaction
SGLT1: Solute carrier family 5 member 1
si-RNA: Small interfering RNA
TC: Total cholesterol
TG: Triglyceride
WGA: Wheat germ agglutinin
α-SMA: α-Smooth muscle actin
β-MHC: β-Myosin heavy chain
