# Supplementary material for: Xinbao Pill ameliorates heart failure via regulating the SGLT1/AMPK/PPARα axis to improve myocardial fatty acid energy metabolism
Source: Chin Med. 2024 Jun 11;19:82. doi: 10.1186/s13020-024-00959-1 (PMC11165817; doi:10.1186/s13020-024-00959-1)
Supplement: Supplementary file 1 — Supplementary material 1. [file 13020_2024_959_MOESM1_ESM.docx]

**Supplemental Material**

**Quantitative real-time PCR (qRT-PCR) assays**

The target genes were validated in ISO-induced NRCMs using qRT-PCR technology. After cell treatment, total RNA was isolated from the cells using Trizol reagent, and mRNA levels were detected using SYBR Green Master Mix from Vazyme (Nanjing, China), with β-actin as the amplification reference.

Data were analysed using the 2^-△△Ct^ method, and primer sequences are listed in Table S1.

**Table S1 The primer sequences of hub genes**

| **Protein** | **Gene** | **Former primer** | **Reverse primer** |
| --- | --- | --- | --- |
| SGLT1 | *Slc5a1* | CCGCTGTTACTGCCACCGATG | CCACCACGAAGTAGATGACGATGAC |
| ANP | *Nppa* | TATCACCCTGGGCTTCTTCCTCG | GGACTAGGCTGCAACAGCTTCC |
| β-MHC | *Myh7* | TGCAGCAGAACCCACCCAAGTTC | GGTGCAGCTGCTGCACTCGCAGA |
| β-actin | *β-actin* | CCAGGGCTGTGTTCCCATCCAT | TGCGACATCGACATCAGGAAG |

Table S2 shows the primer sequences for the three siSGLT1 variants with different efficiencies.

**Table S2 The sequences of si-SGLT1**

| **Name** | **Gene** | **Former primer** | **Reverse primer** |
| --- | --- | --- | --- |
| siSGLT1-1 | *siSlc5a1-1* | CCAUGGACAUCUACACCAA | UUGGUGUAGAUGUCCAUGG |
| siSGLT1-2 | *siSlc5a1-2* | CAUGGAUAAGUACAUGAAA | UUUCAUGUACUUAUCCAUG |
| siSGLT1-3 | *siSlc5a1-3* | GCAAGCGGAUCCAGAUCUA | UAGAUCUGGAUCCGCUUGC |

**Figure S1**

**
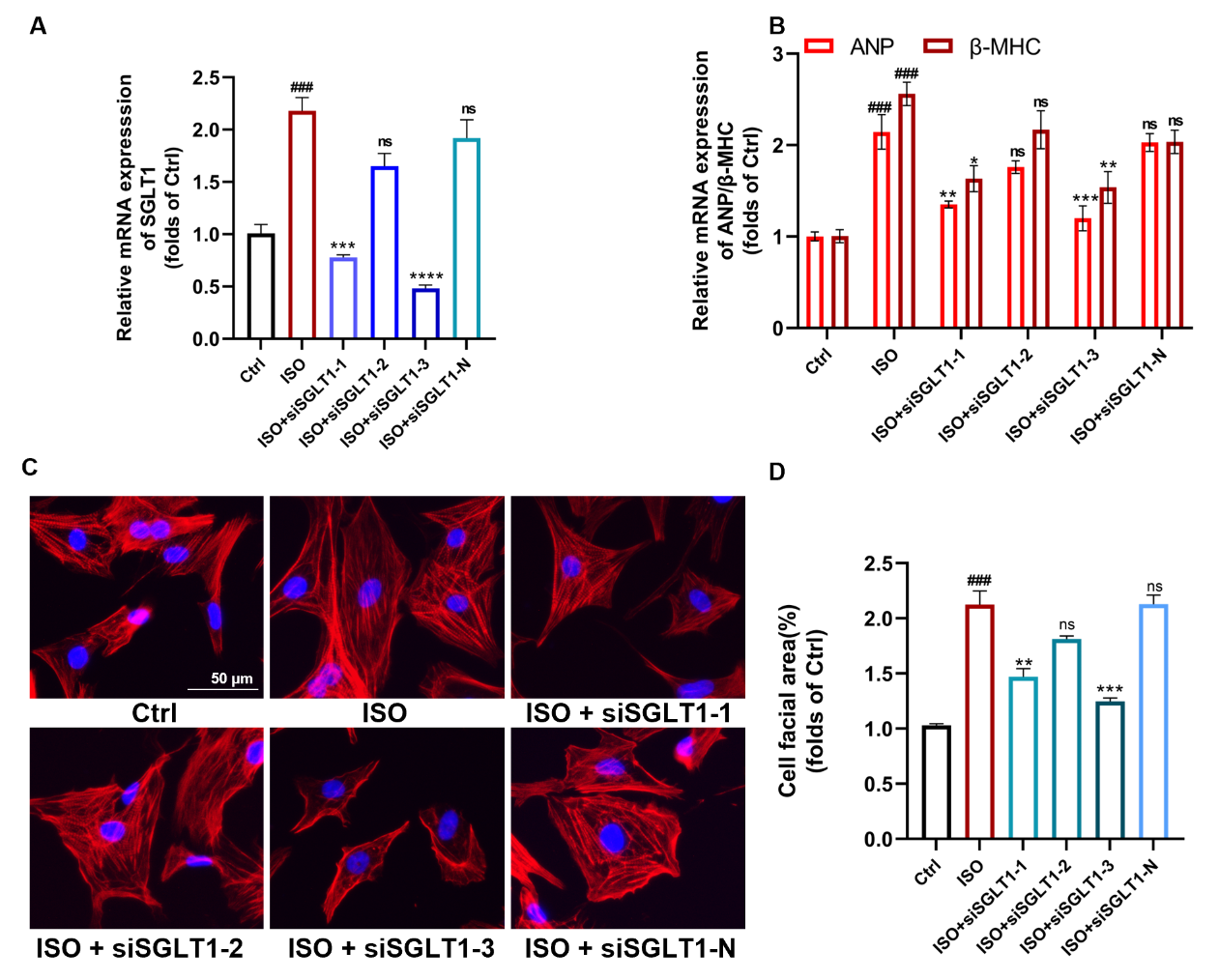
**

**Figure S1. Differential silencing efficiencies of si-SGLT1 and their impact on the cell surface area of ISO-induced NRMCs.**

**(A)** SGLT1 mRNA expression levels in ISO-induced NRCMs. **(B)** ANP/β-MHC mRNA expression levels in ISO-induced NRCMs. **(C-D)** Cell surface area was meticulously evaluated *via* Rhodamine-Phalloidin staining Scale bar: 50 μm.
